# DNA barcoding of reef brittle stars (Ophiuroidea, Echinodermata) from the southwestern Indian Ocean evolutionary hot spot of biodiversity

**DOI:** 10.1002/ece3.3554

**Published:** 2017-11-19

**Authors:** Emilie Boissin, Thierry Bernard Hoareau, Gustav Paulay, J. Henrich Bruggemann

**Affiliations:** ^1^ PSL Research University: EPHE‐UPVD‐CNRS USR 3278 CRIOBE Université de Perpignan Perpignan Cedex France; ^2^ Laboratoire d'Excellence “CORAIL” Papetoai Moorea French Polynesia; ^3^ Molecular Ecology and Evolution Programme Department of Genetics University of Pretoria Pretoria South Africa; ^4^ Florida Museum of Natural History Gainesville FL USA; ^5^ UMR ENTROPIE UR‐IRD‐CNRS Université de La Réunion Sainte‐ClotildeLa Réunion France

**Keywords:** cryptic species, diversification, invertebrates, peripheral areas, species delineation

## Abstract

In anticipation of the current biodiversity crisis, it has become critical to rapidly and accurately assess biodiversity. DNA barcoding has proved efficient in facilitating the discovery and description of thousands of species and also provides insight into the dynamics of biodiversity. Here, we sequenced a portion of the mitochondrial cytochrome c oxidase subunit I (COI) gene from all morphospecies of reef brittle stars collected during a large‐scale biodiversity survey in the southwestern Indian Ocean (SWIO). Three methods of species delineation (Automatic Barcode Gap Discovery, Generalized Mixed Yule Coalescent model, and Bayesian Poisson Tree Processes) showed concordant results and revealed 51 shallow reef species in the region. Mean intraspecific genetic distances (0.005–0.064) and mean interspecific genetic distances within genera (0.056–0.316) were concordant with previous echinoderm studies. This study revealed that brittle‐star biodiversity is underestimated by 20% within SWIO and by >40% when including specimens from the Pacific Ocean. Results are discussed in terms of endemism, diversification processes, and conservation implications for the Indo‐West Pacific marine biodiversity. We emphasize the need to further our knowledge on biodiversity of invertebrate groups in peripheral areas.

## 1 INTRODUCTION

The rapid loss of biodiversity has made rapid and comprehensive biodiversity assessments a high priority (Losos et al., 2013; Plotnick, Smith, & Lyons, 2016; Regnier et al., 2015). A recent survey of 10 well‐known taxa (e.g., mammals, birds, reptiles) in Europe, one of the best studied biogeographic regions in the world, has demonstrated that our current knowledge of species diversity is far from complete, and is biased toward widespread species (Essl, Rabitsch, Dullinger, Moser, & Milasowszky, 2013). This work also revealed that endemics were described on average 79 years later than widespread species and that they are especially likely to be still awaiting discovery (19% estimated undiscovered for endemics compared with 3% for widespread species). Less well‐known groups in underexplored areas are thus especially worthy targets of investigation.

A number of recent studies have demonstrated that brittle stars are promising models for understanding marine diversification (Boissin, Egea, Feral, & Chenuil, 2015; Boissin, Feral, & Chenuil, 2008; Boissin, Hoareau, Feral, & Chenuil, 2008; Hoareau, Boissin, Paulay, & Bruggemann, 2013; Stöhr, Boissin, & Chenuil, 2009). The group includes numerous cryptic species, many with a restricted distribution, especially in peripheral areas (Boissin, Stöhr, & Chenuil, 2011; Hoareau, Boissin, Paulay & Bruggemann, 2013). The role of peripheral endemics in marine diversification has received recent emphasis, with the demonstration that many contribute to diversification through range expansion (Bowen, Rocha, Toonen, Karl, & ToBo, 2013). The southwestern Indian Ocean (SWIO) is a peripheral area of the Indo‐West Pacific (IWP) region and was recently proposed as a potential evolutionary hot spot, defined as an area that is able to maintain as well as generate biodiversity (Hoareau, Boissin, Paulay & Bruggemann, 2013). The northern Mozambique Channel in this area was also identified as a potential long‐term biodiversity refuge (McClanahan, Ateweberhan, Darling, Graham, & Muthiga, 2014; Obura, 2016). Furthermore, the clear‐water Maldives, Seychelles, and southwest Madagascar and more turbid southern Mozambique Channel and Reunion Island are predicted macrorefuges under future warmer conditions (Cacciapaglia & van Woesik, 2015, 2016). Thus, while the SWIO is clearly important both in diversification and as a refuge, our knowledge on biodiversity of the region remains limited.

Field surveys coupled with DNA barcoding have proved useful for rapidly assessing biodiversity (Hebert, Cywinska, Ball, & DeWaard, 2003; Miller, Hausmann, Hallwachs, & Janzen, 2016). An integrative approach to taxonomy (i.e., using morphological characters from both living and preserved specimens, as well as one to several genes) has emerged as a powerful and necessary means for assessing species diversity and species boundaries (Puillandre, Lambert, Brouillet, & Achaz, 2012). Furthermore, assessing species diversity and boundaries based on sequence data has become an active field as a result of the rise in DNA barcode and metabarcode data (Fontaneto, Flot, & Tang, 2015; Pons et al., 2006; Zhang, Kapli, Pavlidis, & Stamatakis, 2013). The Automatic Barcode Gap Discovery method (Puillandre et al., 2012) uses the potential “barcode gap” between intra‐ and interspecific genetic differences to delineate species. Other methods are based on the expected change in the branching pattern of the phylogenetic tree, between phylogenetic (interspecific) and coalescent (intraspecific) processes (Generalized Mixed Yule Coalescent model, Pons et al., 2006; Fujisawa & Barraclough, 2013) or on branch length distributions on the phylogenetic tree (Poisson Tree Processes model, Zhang et al., 2013).

Previous studies have confirmed that DNA barcoding can successfully discriminate echinoderm species (Hoareau & Boissin, 2010; Layton, Corstorphine, & Hebert, 2016; Uthicke, Byrne, & Conand, 2010; Ward, Holmes, & O'Hara, 2008). Our previous work on selected brittle‐star species highlighted how DNA barcoding can reveal evolutionary processes in the SWIO (Hoareau, Boissin, Paulay & Bruggemann, 2013). A recent taxonomic inventory of shallow water brittle stars at Reunion Island (Mascarene, SWIO) yielded 15 new records and suggested the existence of cryptic and endemic species (Boissin, Hoareau, Paulay, & Bruggemann, 2016). To improve our understanding of this still largely understudied group and investigate cryptic and endemic species in the SWIO, we here apply a combination of DNA barcoding and species delineation methods.

## 2 MATERIALS & METHODS

### 2.1 Sampling and taxonomic determination

**Figure 1:**
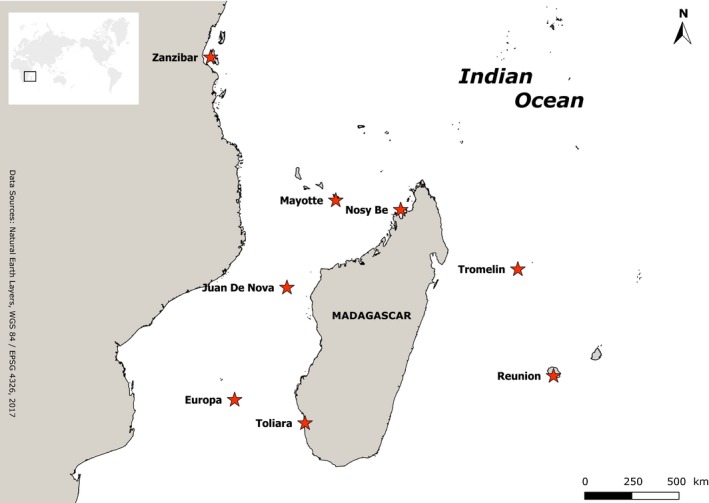
Map of the southwestern Indian Ocean localities where shallow water reef brittle stars were collected for this study

Specimens were collected in the SWIO during 2007–2009, at Reunion, Mayotte, Madagascar (Nosy Be and Toliara), and the Scattered Islands (Figure 1; see Table S1 for details). Animals were anesthetized with MgCl_2_, photographed, and preserved in ethanol for subsequent morphological study and DNA sequencing. Specimens from Zanzibar and Oman (in the Indian Ocean) and from various Pacific localities housed in the Florida Museum of Natural History collection were also included (see Table S1). Additional sequences from related species available on GenBank were included.

Traditional species identifications (i.e., primary species hypotheses, or PSH, see Puillandre et al., 2012) were based on morphological identifications using the regional taxonomic guides of Clark and Rowe (1971) and Cherbonnier and Guille (1978), refined with the subsequent taxonomic literature as available (see Boissin, Hoareau, Paulay & Bruggemann, 2016 for details). A total of 42 nominal SWIO reef‐associated species were included in this study.

### 2.2 Molecular analyses

DNA was extracted from a piece of arm using the DNeasy Kit (Qiagen, Hilden, Germany). The Folmer region of the DNA barcoding gene, cytochrome c oxidase subunit I (COI), was amplified using echinoderm‐specific hybrid primers following the protocol of Hoareau and Boissin (2010). PCR products were verified on 1% agarose gels and sent for sequencing at the Interdisciplinary Center for Biotechnology Research at the University of Florida.

### 2.3 Data analyses

#### 2.3.1 Phylogenetic reconstruction

Sequences were aligned using Mafft online (Katoh, Misawa, Kuma, & Miyata, 2002; Katoh & Standley, 2013). MrAic v.1.4.4 (Nylander, 2004) was used to infer the best model of nucleotide substitution. Phylogenetic reconstructions were performed using the neighbor‐joining and maximum likelihood algorithms in Mega7 (Kumar, Stecher, & Tamura, 2016). Bayesian phylogenetic reconstructions were performed with Beast2 (Bouckaert et al., 2014), with every 100th generation recorded among 10,000,000, a burn‐in of 10%, and the final 10,000 trees summarized using TreeAnnotator. An exponential relaxed clock and a birth–death tree model were used as priors to obtain an ultrametric tree for the Generalized Mixed Yule Coalescent model analyses (see below). tracer v1.6 (Rambaut, Suchard, Xie, & Drummond, 2014) was used to ensure that enough generations were computed (Effective Sample Sizes >200).

#### 2.3.2 Delineation of taxonomic units

Three methods were used to sort sequences into genetic species (secondary species hypotheses, or SSH, see Puillandre et al., 2012). (1) The Automatic Barcode Gap Discovery (ABGD, Puillandre et al., 2012) is a distance method that relies on the gap between the distribution of interspecific and intraspecific genetic distances. ABGD was run online at wwwabi.snv.jussieu.fr/public/abgd/abgdweb.html. Parameters were set at default values, except X (relative gap width), which was set to 1. (2) The Generalized Mixed Yule Coalescent model (GMYC, Pons et al., 2006; Fujisawa & Barraclough, 2013) is a likelihood‐based method for delimiting species by fitting within‐ and between‐species branching models to reconstruct gene trees, using an ultrametric tree (i.e., the Bayesian tree reconstructed using Beast2, see previous section). We used both a single threshold (hereafter referred as GMYCst) and a multiple thresholds (hereafter referred as GMYCmt) method for differentiating between population and phylogenetic processes; these assume that the transition from coalescence to speciation occurs a single or multiple times across the phylogeny. (3) The Bayesian implementation of the Poisson Tree Processes model (bPTP, Zhang et al., 2013) uses a phylogenetic tree and is based on the phylogenetic species concept. The ML tree was used as input. These two last methods were run on the species delimitation web interface (species.h‐its.org, Zhang et al., 2013). The PTP analysis was run for 500,000 MCMC generations, with a thinning value of 100 and a burn‐in of 10%.

#### 2.3.3 Genetic distances

Kimura‐2 parameter distances were computed among specimens within primary (when including specimens from the Pacific) and secondary species hypotheses, as well as among species within each genus using Mega7.

## 3 RESULTS

**Figure 2:**
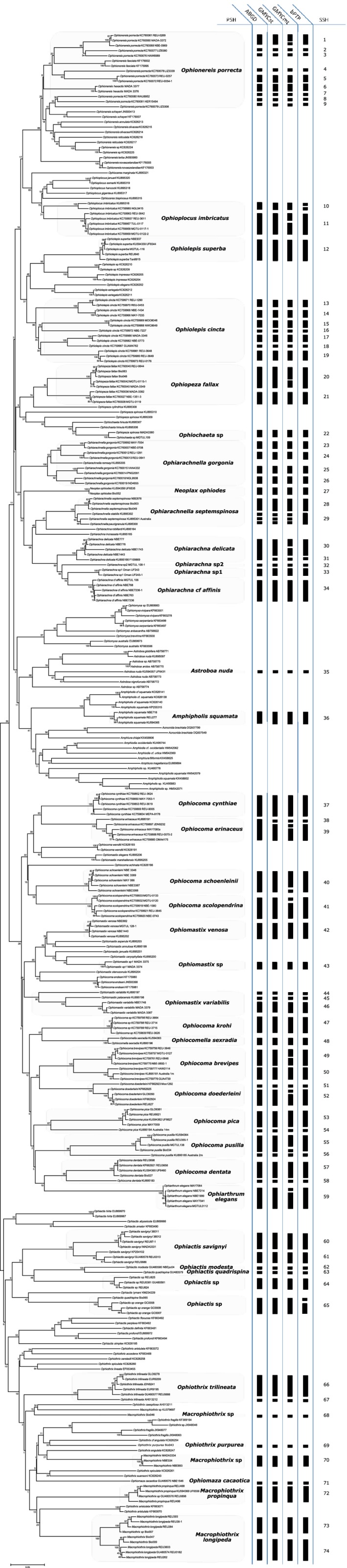
Neighbor‐joining phylogenetic reconstruction based on 300 COI sequences and K2P genetic distances with overlaid results of the three delineation methods. The highlighted species are the 42 PSH: primary species hypothesis (nominal species) collected in the SWIO and the focus of this study; ABGD: results from Automatic Barcode Gap Discovery method (Puillandre et al., 2012); GMYC: species delimitation from Generalized Mixed Yule Coalescent method (Fujisawa & Barraclough, 2013; Pons et al., 2006) using single threshold (GMYCst) or multiple thresholds (GMYCmt); bPTP: species delimitation using Bayesian Poisson Tree Processes method (Zhang et al., 2013); SSH: secondary species hypothesis

A total of 675 sequences were analyzed in this study (Figure S1, Table S1), and a smaller dataset of 300 sequences (retaining representative sequences of each species) was used to graphically represent the results (Figure 2).

### 3.1 Phylogenetic reconstruction and species delineation

Three methods recovered broadly the same number of SSH (Figures 2, S2, S3): 156 SSH in the total data set when using ABGD; 162 SSH using GMYCst; 175 SSH using GMYCmt; and 164 SSH using bPTP. The 42 PSH present in the SWIO resulted in 51 SSH when using ABGD or GMYCst, 62 using GMYCmt, and 52 SSH using bPTP. Considering additional specimens collected in the Pacific for these 42 PSH present in the SWIO resulted in 70 SSH using ABGD, 72 SSH using GMYCst, 83 SSH using GMYCmt, and 73 SSH using bPTP.

As species delineation methods have a tendency to overestimate the number of species present in a dataset, we selected a consensus dataset of species that were delineated by at least three of the four analyses; 74 SSH among the focal taxa were so recovered by at least three of the analyses, including 51 SSH within the SWIO.

Noticeably, the PSH *Ophionereis porrecta* and *Ophiolepis cincta* were composed of multiple lineages. The *Ophiocoma* genus revealed cryptic species between its populations in the Indian and Pacific Oceans.

### 3.2 Genetic distances

Intraspecific K2P genetic distances ranged from 0.005 (*Ophiarachna*) to 0.064 (*Ophionereis*) with a mean of 0.022 (Table 1). Interspecific genetic distances within genera ranged from 0.056 (*Ophiomyxa*) to 0.316 (*Ophiactis*) with a mean of 0.189. The inclusion of Pacific populations substantially increased genetic distances relative to those obtained when considering SWIO specimens only (e.g., intraspecific genetic distance for all *Ophiocoma/Ophiomastix = 0.024* vs *0.010 for Ophiocoma/Ophiomastix* from only the *SWIO*).

**Table 1:** Average K2P genetic distances within species and between species within genera analyzed in this study. Values in parentheses are standard deviations

Genus	K2P distance within species	K2P distance between species
*Astroboa*	–	0.240 (0.030)
*Ophiomyxa*	–	0.056 (0.021)
*Ophiarachna/Ophiarachnella*	0.005 (0.004)	0.083 (0.035)
*Ophiopeza*	0.009 (0.003)	0.205 (0.044)
*Ophiocoma/Ophiomastix*	0.024 (0.020)	0.239 (0.040)
*Ophiocoma/Ophiomastix* SWIO	0.010 (0.005)	0.238 (0.040)
*Ophiactis*	0.034 (0.029)	0.316 (0.056)
*Ophionereis*	0.064 (0.040)	0.174 (0.032)
*Ophionereis* SWIO	0.008 (0.004)	0.172 (0.033)
*Ophioplocus*	0.023 (0.015)	0.120 (0.036)
*Ophioplocus* SWIO	0.007 (0.004)	0.113 (0.042)
*Ophiolepis*	0.012 (0.008)	0.200 (0.035)
*Ophiothrix/Macrophiothrix*	0.052 (0.030)	0.300 (0.079)
Total	0.022 (0.019)	0.189 (0.079)

## 4 DISCUSSION

This study complements the taxonomic and DNA barcoding works published by Stöhr, Conand, and Boissin (2008), Hoareau and Boissin (2010), Hoareau, Boissin, Paulay & Bruggemann (2013), Bollard et al. (2013), Boissin, Hoareau, Paulay & Bruggemann (2016), thus furthering our knowledge of the global diversity of reef brittle stars. The results show that brittle‐star biodiversity is still largely underestimated: by 20% within SWIO and by >40% when including specimens from the Pacific Ocean. Cryptic lineages were uncovered in 6 of 42 PSH in the SWIO and in eight additional PSH when Pacific specimens are also considered. The mean genetic distances within species (2.2%) and between species within genera (18.9%) were of the same order of magnitude to that found in echinoderms in general by Hoareau and Boissin (2010) (1.3% and 20.3%, respectively). The genera *Ophiocoma*,* Ophiomastix,* and *Ophiocomella* were all found to be nonmonophyletic, as also shown by a taxonomic revision of the family Ophiocomidae in preparation (O'Hara TD, unpublished).

### 4.1 Discovery of numerous cryptic species

The three species delimitation methods recovered broadly the same number of SSH. Importantly, the use of different substitution models of evolution did not significantly affect the number of species recovered from the SWIO (Table S2). Also the tree prior in Beast (Yule speciation model, Birth–Death model or coalescent model) or the phylogenetic reconstruction used (NJ or ML) did not affect the results much (Table S2). Noticeably, the GMYC multiple thresholds recovered a higher number of species than all other methods, but this method is known to overestimate the number of delineated species (Fujisawa & Barraclough, 2013). Furthermore, GMYC might have performed poorly, as estimating an ultrametric tree with a single locus on an entire class is not optimal and will tend to compress the coalescent events toward the tips of the tree, thus making closely related species more difficult to distinguish. Nevertheless, it broadly recovered the same number of SSH as the other two methods. Overall, these results emphasize the need to perform all three methods and compare their results to help mitigate their potential drawbacks.

The 42 nominal species sampled in the SWIO revealed 51 SSH within this area (20% increase) and 74 SSH when specimens from the Pacific were included (>40% increase). Among the six PSH that included cryptic lineages within the SWIO, four have lecithotrophic larvae (*Ophiarachnella gorgonia*,* O. porrecta*,* Ophiopeza fallax,* and *O*. *cincta*; see Hoareau, Boissin, Paulay & Bruggemann, 2013). This larval type is associated with limited dispersal capacity compared to species with planktotrophic larvae and could thus facilitate differentiation on small spatial scales (Boissin, Stöhr & Chenuil, 2011).

Cryptic diversity is particularly common in widespread species: Many marine species that were thought to be widespread have been found to include multiple lineages with more restricted geographic ranges (Boissin, Hoareau, & Berrebi, 2011; Boissin, Stöhr & Chenuil, 2011; Dawson, 2005; Murray, 2007; Postaire, Gelin, Bruggemann, & Magalon, 2017; Postaire, Magalon, Bourmaud, & Bruggemann, 2016). For example, *O. cincta*, until recently considered to have an IWP‐wide distribution, is represented by four cryptic lineages in the SWIO alone (Figure 2; Hoareau, Boissin, Paulay & Bruggemann, 2013) and approximately 15 across the IWP (Pineda‐Enriquez, Boissin, & Paulay, 2015). *O. porrecta,* previously considered similarly widespread, includes at least eight cryptic species as revealed in our study. Cryptic lineages are particularly common in peripheral areas, such as the SWIO (e.g., *M. longipeda*,* O. gorgonia*,* O. porrecta*), presumably as a result of peripatric speciation (see 4 in Boissin et al., 2016). Differentiation was also observed between Indian and Pacific Ocean populations of several of the nominal species, as well documented for many groups (Bowen et al., 2016; Gaither & Rocha, 2013; Hubert et al., 2012) and the brittle‐star case is the focus of an ongoing study (Boissin E, Hoareau T, Bruggemann H, Paulay G, unpublished).

Cryptic species can also be the consequence of limited or overlooked morphological differentiation. Several of the new SSH are morphologically distinguishable, but previous studies have either overlooked these differences, or attributed them to intraspecific variability. Among ophiocomids, which include some of the largest and most studied brittle stars, notable recently described species include *Ophiocoma cynthiae* (Benavides‐Serrato & O′Hara, 2008) and *Ophiocoma krohi* (Boissin, Hoareau, Paulay & Bruggemann, 2016; Stöhr, Boissin, & Hoareau, 2013), both widespread and common species.

### 4.2 Endemism, conservation, and dynamics of biodiversity

Studies of diversification that focus only on known, nominal species are problematic, as they likely overlook cryptic lineages involved in diversification. Not recognizing cryptic diversity is also of conservation concern, especially because such lineages are often endemic or rare (Rodrigues & Gaston, 2002; Soltis & Gitzendanner, 1999), and thus tend to be more vulnerable (Essl et al., 2013). Modern conservation practices aim to maximize phylogenetic diversity in the selection of networks of conservation areas (Buerki et al., 2015; Rodrigues & Gaston, 2002). Importantly, biodiversity loss does not yet represent the current impact of humans but rather corresponds to the effects of early to mid‐century pressures (Dullinger et al., 2013), as biodiversity responses to changing environmental forcing on species are often characterized by considerable time lags (Essl et al., 2013). Focus on charismatic megafauna, such as vertebrates, has meant that our understanding of human impacts on more diverse groups, such as invertebrates, is inadequate (Régnier et al., 2015). Biodiversity inventories and integrative taxonomic studies, such as presented here, provide the needed comprehensive and rapid assessments of missed diversity.

Finally, the present study explored only a limited number of specimens, with samples mostly concentrated in the SWIO. The Indo‐Pacific region is already the biogeographic area holding the highest brittle‐star species diversity (Stöhr et al., 2013), but its diversity is likely largely underestimated. Broader efforts, especially those that include samples from the Indo‐Australian Archipelago hot spot of biodiversity, but also from regions with high endemism (e.g., Hawaii, Marquesas and the Red Sea), are likely to result in the discovery of substantial additional cryptic diversity. This will in turn refine our perception of the marine biodiversity dynamics of the Indo‐Pacific region.

## DATA ACCESSIBILITY

GenBank accession numbers of all the COI sequences used in this study are listed in Table S1 (MF989246‐MF989437).

## AUTHOR CONTRIBUTION

EB designed the study, conducted the analyses, and drafted the manuscript. All authors participated in sample collections, read, and commented on the manuscript.

## CONFLICT OF INTEREST

None declared.
