# Supplementary material for: DNA barcoding of reef brittle stars (Ophiuroidea, Echinodermata) from the southwestern Indian Ocean evolutionary hot spot of biodiversity
Source: Ecol Evol. 2017 Nov 19;7(24):11197–203. doi: 10.1002/ece3.3554 (PMC5743570; doi:10.1002/ece3.3554)

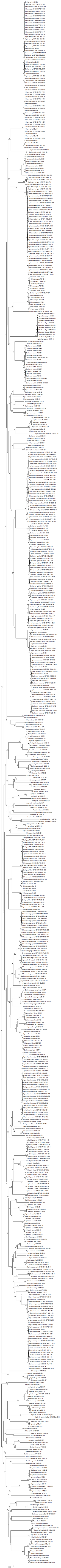

Supplement: Supplementary file 1 [file ECE3-7-11197-s001.pdf]
